# Surface Electromyography Normalization Affects the Interpretation of Muscle Activity and Coactivation in Children With Cerebral Palsy During Walking

**DOI:** 10.3389/fneur.2020.00202

**Published:** 2020-04-17

**Authors:** Yngvild Gagnat, Siri Merete Brændvik, Karin Roeleveld

**Affiliations:** ^1^Clinic for Orthopaedics, Rheumatology and Skin Diseases, Orthopaedic Research Center, St. Olavs University Hospital, Trondheim, Norway; ^2^Department of Neuromedicine and Movement Science, Faculty of Medicine and Health, Norwegian University of Science and Technology, NTNU, Trondheim, Norway; ^3^Clinical Services, St. Olavs University Hospital, Trondheim, Norway

**Keywords:** surface electromyography, muscle activity, coactivation, cerebral palsy, gait, amplitude normalization

## Abstract

Investigating muscle activity and coactivation with surface electromyography (sEMG) gives insight into pathological muscle function during activities like walking in people with neuromuscular impairments, such as children with cerebral palsy (CP). There is large variation in the amount of coactivation reported during walking in children with CP, possibly due to the inconsistent handling of sEMG and in calculating the coactivation index. The aim of this study was to evaluate how different approaches of handling sEMG may affect the interpretation of muscle activity and coactivation, by looking at both absolute and normalized sEMG. Twenty-three ambulatory children with CP and 11 typically developing (TD) children participated. We conducted a three-dimensional gait analysis (3DGA) with concurrent sEMG measurements of tibialis anterior, soleus, gastrocnemius medialis, rectus femoris, and hamstring medialis. They walked barefoot at a self-selected, comfortable speed back and forth a 7-m walkway. The gait cycle extracted from the 3DGA was divided into six phases, and for each phase, root mean square sEMG amplitude was calculated (sEMG-RMS-abs), and also normalized to peak amplitude of the linear envelope (50-ms running RMS window) during the gait cycle (sEMG-RMS-norm). The coactivation index was calculated using sEMG-RMS-abs and sEMG-RMS-norm values and by using two different indices. Differences between TD children's legs and the affected legs of children with CP were tested with a mixed model. The between-subject muscle activity variability was more evenly distributed using sEMG-RMS-norm; however, potential physiological variability was eliminated as a result of normalization. Differences between groups in one gait phase using sEMG-RMS-abs showed opposite differences in another phase using sEMG-RMS-norm for three of the five muscles investigated. The CP group showed an increased coactivation index in two out of three muscle pairs using sEMG-RMS-abs and in all three muscle pairs using sEMG-RMS-norm. These results were independent of index calculation method. Moreover, the increased coactivation indices could be explained by either reduced agonist activity or increased antagonist activity. Thus, differences in muscle activity and coactivation index between the groups change after normalization. However, because we do not know the truth, we cannot conclude whether to normalize and recommend incorporating both.

## Introduction

Surface electromyography (sEMG) is used to measure muscle activity and may be used clinically to investigate muscle function during activities such as walking in conditions affecting the neuromuscular system (1). In children with cerebral palsy (CP), three-dimensional gait analysis (3DGA) with simultaneous sEMG measurements is often conducted to get insight into muscle activity as part of treatment prescriptions and evaluation of treatment effect. Cerebral palsy, the most common cause of physical disability in childhood, is characterized by insufficient motor activity such as reduced muscle strength and poor balance, but also increased motor activity such as spasticity and excessive muscle coactivation (2, 3). Those features of children with CP may impair function in general and gait in particular. Compared to typically mature gait, children with CP have shown deviations in different gait phases and greater physiological variability during walking (4, 5).

Muscle coactivation, defined as simultaneous activity of agonist and antagonist muscles crossing the same joint, is a normal motor control strategy to increase joint stability and coordination (6, 7). During complex tasks, such as walking, coactivation occurs prominently at certain phases during the gait cycle, ensuring stability and allowing efficient walking (8). Excessive and/or prolonged coactivation, however, may cause inefficient movements by reducing flexibility and adaptability and increasing the loading of the joints, and thus, energy cost (6, 7, 9, 10). Therefore, a main treatment goal for ambulatory children with CP is to make walking easier, through, for example, normalizing altered muscle activity and coactivation (11). However, the role of the increased coactivation in children with CP has been questioned in several studies, and the findings are conflicting (9, 12–14). Coactivation may be quantified using a coactivation index, which is a value calculated to represent the amount of coactivation between the given muscles. Potential challenges when it comes to investigating and interpreting muscle activity and coactivation, which also may explain the diversity of findings, could be choice of approach for handling the sEMG data and calculations of the coactivation index (15).

The amplitude of the sEMG represents the number of motor units recruited and their firing rate and pattern (16). However, the amplitude is affected by several other factors, such as the size, shape, and material of the electrode and the distance between the electrodes and the active muscle tissue, largely determined by the subcutaneous fat layer, causing nonphysiological between-subject variability. To adjust for this variability and allow for comparison between participants, sEMG signals are commonly normalized to a standard value, usually peak sEMG obtained during a maximal voluntary isometric contraction (MVIC) (15). Children, and especially children with neurological conditions, such as CP, may have difficulties in performing an MVIC because it is challenging to voluntarily produce maximal muscle activation (12, 13, 17). In this case, normalizing to peak sEMG obtained during the specific task to be evaluated, that is during walking, is considered a feasible and appropriate approach (15, 18). However, the peak sEMG during walking may occur at different phases of gait for typically developing (TD) children and children with CP, for example, because of reduced muscle strength or spasticity. This may have consequences for the normalized sEMG in the other phases of gait, which in turn could affect the interpretation of muscle activity. Therefore, in people with neurological conditions, it is also suggested to not normalize the sEMG data (13, 19–22).

The different ways of normalizing sEMG data are used interchangeably when investigating muscle coactivation. Although the majority of the studies in the literature usually normalize the sEMG data prior to calculating the coactivation index (9, 12, 13, 23, 24), using absolute data has been suggested to be beneficial because it prevents unnecessary data transformation (19). Calculations are often done using a ratio, and therefore, normalizing the sEMG data prior may normalize the data twice. In addition, Lamontagne et al. (20) argue for using absolute values when calculating the coactivation index in populations with weak muscles, because the sEMG values could be low during walking, and normalizing to a percent of a peak value before coactivation index calculations in such cases may lead to an overstated index.

Similar to this lack of standardization of sEMG normalization, different indices are used for calculating the coactivation index. For instance, the muscle activity of the antagonist could be compared either to the muscle activity of the agonist only or to the total muscle activity of both the agonist and the antagonist (25). Comparisons between studies are difficult, and it is challenging to form an overall picture of the mechanism when different approaches are used (15).

Therefore, the overall aim of this article was to investigate the effect of sEMG normalization on the interpretation of muscle activity and coactivation. The specific aim was to evaluate the between-subject variability and group differences between the healthy legs of TD children and the affected legs of ambulatory children with CP. In addition, differences between two indices used for calculating the coactivation index were examined.

## Materials and Methods

This is a retrospective cross-sectional study, based partly on baseline data from an ongoing randomized controlled trial (26) and partly on clinical data from regular outpatient follow-up at St. Olavs University Hospital, Trondheim, Norway.

### Participants

In total, 23 ambulatory children diagnosed with unilateral or bilateral spastic CP and classified with Gross Motor Function Classification System level I or II were included in this study. Ages ranged between 6 and 17 years. Exclusion criteria were botulinum toxin A treatment in the lower limb muscles in the preceding last 3 months, and surgery in the legs in the preceding 24 months prior to inclusion. Eleven typical developing (TD) children, within the same age range, were included as reference. Participant characteristics are presented in Table 1. The study was approved by the Regional Committee for Medical and Health Research Ethics in Middle Norway (REK Central), and a written consent in accordance with the Declaration of Helsinki was signed by the parents or guardians prior to participation.

**Table 1:** Descriptive data presented as number (n) or as mean ± standard deviation (SD) for the different groups.

	**TD**	**CP**
N	11	23
Unilateral right/left/bilateral (n)	—	9/8/6
GMFCS I/II (n)	—	17/6
Gender, female/male (n)	7/4	7/16
Age, years (mean ± SD)	9.4 ± 1.3	11.7 ± 3.1
Height, cm (mean ± SD)	136.4 ± 9.3	147.7 ± 18.1
Weight, kg (mean ± SD)	33.6 ± 8.3	42.1 ± 18.2
Left leg length, cm (mean ± SD)	70.6 ± 6.4	77.2 ± 9.9
Right leg length, cm (mean ± SD)	70.9 ± 6.4	77.5 ± 9.7

*TD, typically developing children; CP, children with cerebral palsy; GMFCS, Gross Motor Function Classification System*.

### Procedure and Equipment

Walking was assessed using 3DGA (Vicon Motion Systems, Ltd., Oxford, UK). Ten cameras with a sampling frequency of 200 Hz and three AMTI force plates (Watertown, MA, USA), with a sampling frequency of 1,000 Hz were positioned along a 7-m walkway. Sixteen reflective markers were placed on anatomical landmarks on the lower limbs, according to the Vicon Plug-in-Gait model (27). Participants were instructed to walk barefoot back and forth the walkway at a self-selected, comfortable walking speed. A minimum of three trials with at least two clean foot strikes on the force plates for each leg were obtained.

During the 3DGA, concurrent sEMG of m. tibialis anterior (TA), m. soleus (SOL), m. gastrocnemius medialis (GM), m. rectus femoris (RF), and m. hamstring medialis (HM), was recorded bilaterally using wireless sEMG (Myon AG, Schwarzenberg, Switzerland). Skin preparation and sEMG electrode placement were done according to the SENIAM (Surface Electromyography for the Non-Invasive Assessment of Muscles) guidelines (28). The sEMG recordings were amplified by a 1,000 gain with a sampling frequency at 1,000 Hz.

### Data Analysis

From the TD children and the children with bilateral CP, both legs were included in the analysis. From the children with unilateral CP, only the affected leg was included. The healthy legs from TD children and the affected legs from children with CP henceforth will be referred to as TD and CP, respectively.

Nexus software (Oxford Metrics, Oxford, UK) was used to process kinematic data, define gait cycles, detect events, and calculate the spatiotemporal parameters walking speed (m/s), cadence (steps/min) and step length (m), and export c3d-files with data from the 3DGA. Raw sEMG signals were visually inspected for artifacts and noise using Myon ProEMG (Myon AG, Baar, Switzerland). A customized MATLAB program (R2018b; MathWorks, Inc., Natick, MA, USA), written using the Biomechanical Toolkit (Btk Development Core Team, version 0.3.0.), was used for processing the c3d-files.

Walking speed, cadence, and step length were normalized to leg length (m) as in Hof (29), using the following equations:

$$\begin{array}{l} Normalized\;walking\;speed=speed/\surd \left(leg\;length\times 9.81m/s^{2}\right)\\ \;Normalized\;cadence=cadence/\surd \left(leg\;length\times 9.81m/s^{2}\right)\\ \;Normalized\;step\;length=step\;length/leg\;length \end{array}$$

**Figure 1:**
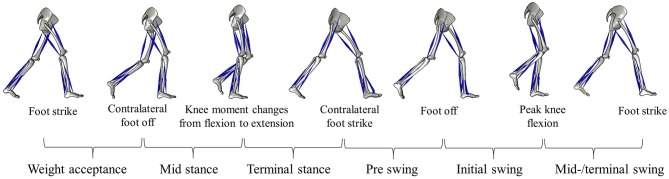
Illustration of a complete gait cycle with the right leg's events, separating the six gait phases: weight acceptance, midstance, terminal stance, preswing, initial swing, midswing/terminal swing.

The gait cycle was divided into six phases based on detected events (Figure 1). The first of these phases was the weight acceptance phase, lasting from ipsilateral foot strike to contralateral foot off. This was followed by midstance, continuing to the point where ipsilateral knee moment changed from external flexion to extension. If this change did not occur, as the external knee moment was continuously in flexion, the mean timing of this event for the equal leg in the respective group was used (i.e., healthy leg in TD/affected leg in CP unilateral or CP bilateral). From this event, terminal stance started and continued to the contralateral foot strike, before preswing lasting to ipsilateral foot off. Then initial swing started and continued to ipsilateral peak knee flexion, preceding midswing/terminal swing lasting to ipsilateral foot strike.

Hip flexion/extension, knee flexion/extension, and ankle dorsi/plantarflexion were time-normalized to the gait cycle. The angles at each percentage of the gait cycle were estimated by using a spline fit.

The raw sEMG data were band-pass filtered using an eighth-order Butterworth filter with cutoff frequency at 30 and 300 Hz. After visually inspecting the data, for all sEMG channels and each percentage of the gait cycle, an sEMG root mean square (RMS) value was calculated with a window of 50 ms. For each sEMG channel, the highest RMS value (peak RMS) was obtained and used for normalization. In addition, the RMS of each sEMG channel was calculated for each of the six gait phases as defined above and illustrated in Figure 1. To evaluate the effect of normalization, both absolute sEMG-RMS amplitudes (μV) and normalized to the peak RMS obtained during the complete gait cycle were included in the analyses, henceforth referred to as sEMG-RMS-abs and sEMG-RMS-norm, respectively.

After visually inspecting the data and assurance of low intrasubject variability, spatiotemporal gait parameters, kinematics, and sEMG-RMS amplitudes were averaged over the included trials to obtain each leg's mean value.

The coactivation index was calculated for all six gait phases, across three muscle pairs (TA/GM, TA/SOL, and RF/HM) using the following two indices (1, 8):

$$\begin{array}{l} CoA1=2*\frac{sEMG_{antagonist}}{sEMG_{agonist}+sEMG_{antagonist}}*100\\ CoA2=\frac{sEMG_{antagonist}}{sEMG_{agonist}}*100 \end{array}$$

In coactivation index 1 (CoA1), the antagonist activity was normalized in relation to the mean total muscle activity and multiplied by two to counterbalance the activity of the agonist (8). In coactivation index 2 (CoA2), the antagonist was expressed as a percentage of agonist muscle activity only. For both indices, a coactivation index of 100% represents equal activity of the agonist and antagonist muscles, whereas 0% represents solely agonist activation.

The definitions of agonist and antagonist muscles are often based on the magnitude of the sEMG amplitude, where the higher signal is assigned to the agonist (19). However, this presumption may not hold in a population with altered muscle activity, especially during complex tasks such as walking. It is therefore necessary to allow for changes in the agonist and antagonist roles throughout the gait cycle, based on the biomechanical function of the muscles around the knee and ankle (19, 30). For TA/GM and TA/SOL coactivation, TA was defined as agonist during the weight acceptance phase, working to control lowering of the foot and during initial and midswing/terminal swing, lifting the foot from the ground, and ensuring foot clearance. Gastrocnemius medialis and SOL were defined as agonists during midstance, terminal stance, and preswing, where they are main contributors to stabilize, control for ankle dorsiflexion, and prepare for foot off. For RF/HM coactivation, RF was defined as agonist during weight acceptance, limiting the magnitude of flexion occurring as the foot strikes the ground, midstance and terminal stance, initiating knee extension, and preswing, controlling for knee flexion. Hamstring medialis was defined as agonist during initial and midswing/terminal swing, initiating knee flexion and preparing for foot clearance, and controlling for knee extension and decelerating the swinging leg, respectively.

### Statistical Analysis

Statistical analyses were carried out using MATLAB (R2018b; MathWorks, Inc.). From the kinematic data (hip, knee, and ankle joint angles) and sEMG-RMS amplitudes per percentage of the gait cycle, group (TD and CP) averages and 95% confidence interval (CI) were calculated and displayed. For each percentage of the gait cycle, the CP group data were defined as different from TD when the average of the group did not overlap with the 95% CI of the other group.

Between-group (TD vs. CP) differences in the spatiotemporal gait parameters, sEMG-RMS amplitudes, and coactivation indices for the six different gait phases were tested using linear mixed models. The spatiotemporal gait parameter, muscle or coactivation muscle pair of interest was set as dependent variable, leg as fixed effect, and subject as random effect. Normality of residuals was checked by visual inspection of QQ plots. Where residuals were not normally distributed, analysis was additionally carried out using log-transformed data. In case of similar results, *p* values from the analysis with non–log-transformed data, henceforth referred to as original analysis, are presented. Mean with 95% CI values for TD and mean with 95% CI deviations of the CP group from TD are retrieved from the original analyses in all cases. Significance was set at *p* < 0.05, and trends are reported where *p* < 0.1.

## Results

**Figure 2:**
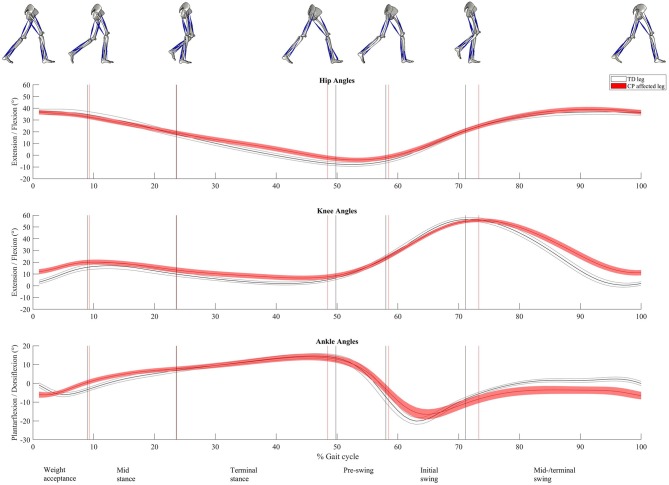
Sagittal-plane joint kinematics. At the top hip extension/flexion; in the middle, knee extension/flexion; and at the bottom, ankle plantarflexion/dorsiflexion. Time normalized to 0 to 100% of the gait cycle. Presented as mean (solid line) with 95% confidence interval (shaded area). The vertical lines represent the mean timing of the different events dividing the gait cycle into six gait phases (named at the bottom line). Gray color is used for the healthy legs of typically developing children and red for the affected legs of children with CP. Illustrations of the right leg's events, separating the different phases, are seen at the top.

**Figure 3:**
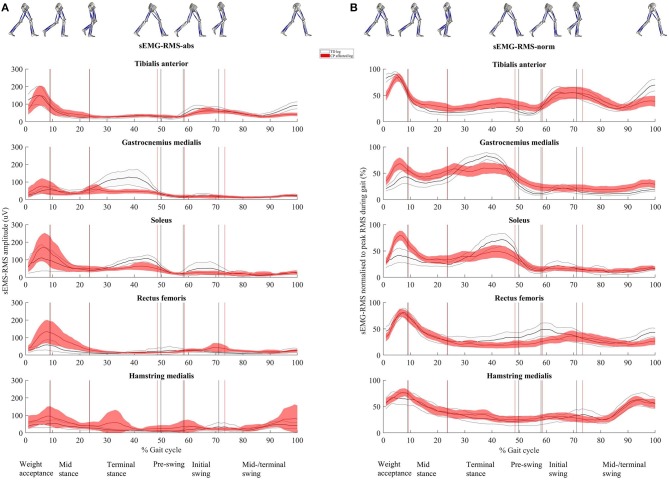
**(A)** Root mean square (RMS) surface electromyographic (sEMG) amplitude (μV, sEMG-RMS-abs) and **(B)** RMS of sEMG normalized to peak RMS obtained during the gait cycle (%, sEMG-RMS-norm) for the five muscles. Time normalized to 0 to 100% of the gait cycle. Presented as mean (solid line) with 95% confidence interval (shaded area). The vertical lines represent the mean timing of the different events dividing the gait cycle into the six gait phases (named in the bottom line). Gray color is used for the healthy legs of typically developing children and red for the affected legs of children with CP. Illustrations of the right leg's events, separating the different phases, are seen at the top.

Twenty-two healthy legs from TD children and 29 affected legs from children with CP were included for the analyses. Spatiotemporal gait characteristics are presented in Table 2. The CP group had significantly lower normalized walking speed and normalized cadence (*p* < 0.01) and shorter normalized step length (*p* = 0.02) compared to the TD group. The percentage time in single support (midstance and terminal stance combined) was significantly shorter for the CP group (*p* < 0.04), whereas there was no difference between the groups in percentage time in double support (weight acceptance and preswing combined, *p* = 0.3). The relative duration of the different gait phases varied between the groups, where the CP group had significantly longer time in preswing and initial swing compared to TD (*p* = 0.002 and *p* < 0.001). The differences in mean timing of the detected events separating the gait phases are illustrated as vertical lines in Figures 2, 3. The CP group had increased hip flexion of ~7 degrees during terminal stance and preswing (Figure 2). During the majority of the gait cycle, the CP group had increased knee flexion, except for during preswing and initial swing. The difference was largest (~10 degrees) during weight acceptance and midswing/terminal swing. The CP group had ~6 degrees plantarflexion at foot strike, while the TD group was in a neutral position. This was also seen during midswing/terminal swing. However, late in the weight acceptance phase, during midstance and start of the initial swing, the CP group had increased dorsi flexion of about the same size.

**Table 2:** Spatiotemporal gait parameters of the healthy legs of typically developing children (TD) and the deviation of the affected legs of children with cerebral palsy (CP) from TD, presented as mean with 95% confidence interval (CI).

	**TD**			**Deviations of CP from TD**		
	**Mean**	**95% CI**		**Mean**	**95% CI**	
Normalized walking speed	0.46	0.43	0.49	**−0.08**	**−0.11**	**−0.05**
Normalized cadence	48.7	45.0	52.4	**−5.7**	**−10.3**	**−1.2**
Normalized step length	0.81	0.77	0.86	**−0.11**	**−0.16**	**−0.06**
Time in single support (%)	40.7	39.7	41.8	**−1.36**	**−2.7**	**−0.1**
Time in double support (%)	17.9	16.2	19.7	1.1	−1.1	3.3

*Significant group differences (p < 0.05) presented in bold*.

### Effect of Normalization on Muscle Activity

Figure 3 shows muscle activity during the complete gait cycle for TA, GM, SOL, RF, and HM using sEMG-RMS-abs and sEMG-RMS-norm for the TD and CP groups. In both groups, the average gait pattern was very similar when presented as sEMG-RMS-abs or sEMG-RMS-norm, because the gait phases with high and low amplitudes hardly changed (Figure 3). However, it seems that the between-subject variability (thickness of the shaded area) in both the TD and CP groups is more evenly distributed during the gait cycle after normalization than before (Figure 3). However, for TA, GM, SOL, and RF, the between-subject variability is less from terminal stance phase and throughout the gait cycle prior to normalization.

The residuals from the linear mixed model either were not normally distributed or had an outlier for the majority (26 of 30) of the sEMG-RMS-abs variables and for 13 of 30 variables of the sEMG-RMS-norm. These variables were log transformed, and the results were similar to the original analysis for 35 of the 39 variables in total. The four variables with changed results are marked with α in Table 3. One variable (HM sEMG-RMS-abs during preswing) had an outlier that remained following log transformation. We conducted the analysis both with and without this outlier, and it did not change the statistical results; thus, results from the original analysis are presented. Table 3 shows gait phase averaged sEMG-RMS-abs and sEMG-RMS-norm amplitudes for the six gait phases for the TD group and for the deviation of the CP group from TD.

**Table 3:** Muscle activity for healthy legs of typically developing children (TD) during six gait phases and the deviation of the affected legs of children with cerebral palsy (CP) from TD, presented as mean with 95% confidence interval (CI).

	**(A) sEMG-RMS-abs (μV)**						**(B) sEMG-RMS-norm (%)**					
	**TD**			**Deviation of CP from TD**			**TD**			**Deviation of CP from TD**		
**Phases**	**Mean**	**95% CI**		**Mean**	**95% CI**		**Mean**	**95% CI**		**Mean**	**95% CI**	
**TIBIALIS ANTERIOR**												
Weight acceptance^β^	150.8	85.0	216.5	−10.3	−94.6	74.0	95.6	84.6	106.6	*−13.1*	*−27.3*	*1.0*
Midstance	59.1	33.2	85.0	1.3	−32.3	35.0	40.9	29.2	52.6	−1.4	−16.7	13.8
Terminal stance	37.3	28.3	46.2	−3.6	−15.0	7.7	32.0	20.9	43.0	1.8	−12.4	15.9
Preswing	27.0	17.9	36.1	5.8	−6.3	17.8	20.3	12.9	27.7	**9.7**	**0.1**	**19.4**^α^
Initial swing^β^	81.8	62.3	101.2	–**16.9**	–**42.1**	**8.3**^α^	64.8	50.3	79.3	−7.3	−26.1	11.5
Midswing/terminal swing^β^	74.2	61.7	86.7	**−28.1**	**−44.4**	**−11.9**	59.5	47.7	71.2	**−16.0**	**−31.4**	**−0.5**
**GASTROCNEMIUS MEDIALIS**												
Weight acceptance	51.4	4.2	98.6	18.3	−43.8	80.3	42.5	28.9	56.2	**21.0**	**3.3**	**38.7**
Midstance^β^	64.7	33.6	95.9	−3.2	−43.7	37.2	49.6	39.0	60.1	5.6	−8.2	19.3
Terminal stance^β^	112.4	87.2	137.6	**−53.9**	**−86.5**	**−21.2**	81.5	71.0	92.1	**−18.2**	**−32.2**	**−4.1**
Preswing^β^	40.8	16.5	65.1	−15.9	−46.6	14.9	25.1	14.5	35.8	*6.0*	–*7.6*	*19.6*
Initial swing	39.5	24.4	54.7	–**22.0**	–**41.3**	–**2.8**	33.0	21.8	44.2	−10.7	−25.4	4.1
Midswing/terminal swing	26.3	16.2	36.5	−6.3	−19.4	6.8	19.5	14.1	24.9	6.9	−0.3	14.1
**SOLEUS**												
Weight acceptance	107.9	16.7	199.1	42.7	−70.4	155.8	43.9	32.1	55.8	**26.3**	**10.7**	**41.8**
Midstance^β^	85.3	39.1	131.5	−2.9	−60.4	54.6	43.7	33.7	53.7	2.8	−10.4	16.1
Terminal stance^β^	96.1	78.9	113.4	**−38.6**	**−61.1**	**−16.1**	67.1	52.3	81.9	*−18.1*	*−37.3*	*1.1*
Pre–swing^β^	36.7	22.6	50.9	−11.0	−28.7	6.7	25.7	14.6	36.9	−4.6	−18.6	9.4
Initial swing	66.1	36.3	95.9	**−41.3**	**−78.2**	**−4.4**	32.7	21.3	44.1	**−14.8**	**−29.4**	**−0.2**
Mid–/ terminal swing	30.9	18.2	43.6	−6.6	−22.9	9.7	18.8	13.4	24.2	−1.7	−8.8	5.4
**RECTUS FEMORIS**												
Weight acceptance^β^	55.0	9.9	100.1	**55.6**	**−2.8**	**113.9**^α^	78.3	68.7	87.9	−6.8	−19.2	5.6
Midstance^β^	42.6	2.4	82.8	*55.2*	*4.2*	*106.2*^α^	61.3	49.3	73.3	−5.0	−20.4	10.3
Terminal stance^β^	24.0	11.9	36.1	0.3	−15.3	15.8	39.4	30.1	48.6	**−14.7**	**−26.6**	**−2.9**
Preswing^β^	40.2	13.8	66.5	−20.2	−54.9	14.4	45.7	34.3	57.1	**−23.5**	**−38.5**	**−8.5**
Initial swing	28.8	12.1	45.6	12.9	−8.7	34.6	53.6	41.8	65.4	**−15.8**	**−31.2**	**−0.3**
Midswing/terminal swing	23.5	14.1	32.9	3.3	−8.7	15.3	42.0	32.0	52.1	**−13.7**	**−26.7**	**−0.7**
**HAMSTRING MEDIALIS**												
Weight acceptance	62.3	28.6	95.9	18.4	−25.6	62.5	86.7	73.1	100.3	−10.2	−28.2	7.8
Midstance	49.0	14.2	83.7	17.9	−26.7	62.6	62.8	52.6	73.1	−6.1	−19.7	7.5
Terminal stance	24.2	−5.5	53.8	22.6	−16.4	61.6	40.0	29.7	50.2	−0.3	−13.6	13.0
Preswing	15.3	−4.2	34.8	15.6	−10.3	41.4	30.5	19.9	41.1	−0.9	−14.4	12.6
Initial swing^β^	38.8	10.8	66.9	−7.9	−43.5	27.7	40.3	29.4	51.2	−5.8	−19.9	8.2
Midswing/terminal swing^β^	49.9	16.6	83.2	6.8	−36.7	50.3	59.9	51.1	68.6	−8.0	−19.6	3.6

*Muscle activity presented as (A) RMS of sEMG amplitude (μV, sEMG-RMS-abs) and (B) RMS of sEMG normalized to peak RMS obtained during the gait cycle (%, sEMG-RMS-norm). Negative values indicate lower values for the CP group compared to TD. Significant group differences (p < 0.05) presented in bold and p < 0.1 presented in italic*.

α *Analysis conducted on log-transformed data*.

β *Gait phases where the given muscle is defined as agonist. RMS, root mean square; sEMG, surface electromyography*.

For sEMG-RMS-abs amplitudes (column A, Figure 3 and Table 3), in all muscles, both the TD and CP groups had similar values during at least four of the six gait phases. Tibialis anterior was, however, significantly reduced for the CP group during initial and midswing/terminal swing (*p* = 0.05 and *p* = 0.001, respectively). Gastrocnemius medialis and SOL were significantly reduced for the CP group during terminal stance (*p* = 0.002 and *p* = 0.001, respectively) and initial swing (*p* = 0.03 for both). Rectus femoris was significantly increased for the CP group during weight acceptance (*p* = 0.04). During midstance, this increase was borderline significant (*p* = 0.08). Although the average HM amplitude for the CP group was above the TD group during almost the whole gait cycle, no significant group differences were found in this muscle.

For sEMG-RMS-norm (column B, Figure 3 and Table 3), the CP group showed similar amplitudes as the TD group in only two to three of the six gait phases, except for HM where no significant group differences were observed. Tibialis anterior was borderline significantly reduced for the CP group compared to the TD group during weight acceptance phase (*p* = 0.07) and significantly reduced during midswing/terminal swing (*p* = 0.04). During preswing, TA was significantly increased for the CP group (*p* = 0.05). Gastrocnemius medialis and SOL were significantly increased for the CP group during weight acceptance phase (*p* = 0.02 and *p* = 0.001, respectively). During terminal stance, GM was reduced for the CP group (*p* = 0.01), and there was a trend toward a reduction in SOL (*p* = 0.06). During initial swing, SOL was significantly reduced in the CP group (*p* = 0.05). Rectus femoris was significantly reduced for the CP group during terminal stance (*p* = 0.02), preswing (*p* = 0.003), initial (*p* = 0.05), and midswing/terminal swing (*p* = 0.04).

### Effect of Normalization on Calculations of the Coactivation Index

**Figure 4:**
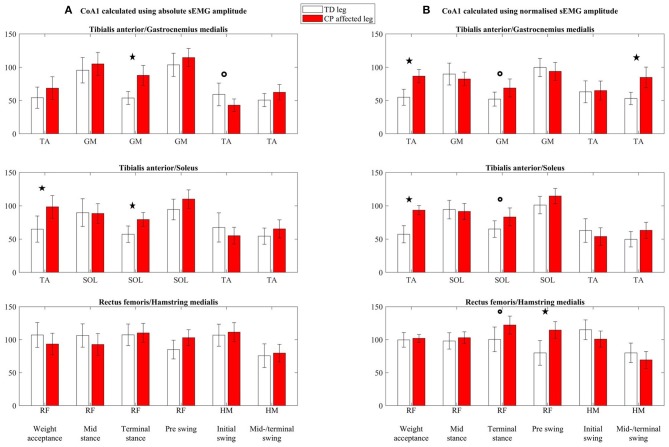
Coactivation index 1 (CoA1) calculated using **(A)** absolute sEMG-RMS amplitudes (CoA1-abs) and **(B)** using normalized sEMG-RMS amplitudes (CoA1-norm), presented as mean with 95% confidence interval. Calculated for three coactivation muscle pairs: tibialis anterior/gastrocnemius medialis, tibialis anterior/soleus, and rectus femoris/hamstring medialis, for each of the six gait phases (named in the bottom line). The agonist in the coactivation index muscle pair is indicated for each gait phase below each subplot. TA, tibialis anterior; GM, gastrocnemius medialis; SOL, soleus; RF, rectus femoris; HM, hamstring medialis. ^*^Significant group differences (*p* < 0.05), °*p* < 0.1.

**Figure 5:**
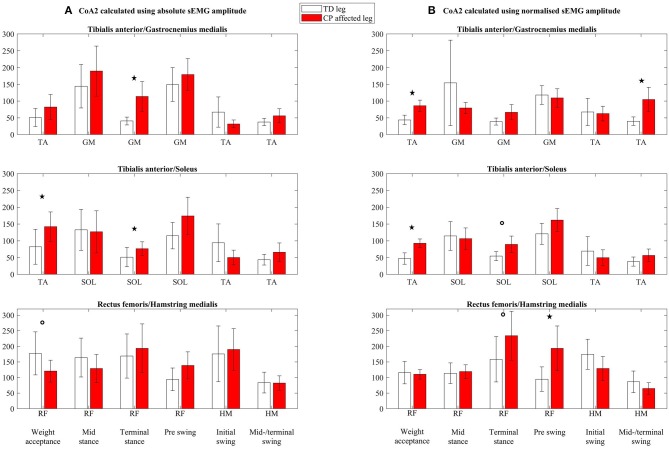
Coactivation index 2 (CoA2) calculated using **(A)** absolute sEMG-RMS amplitudes (CoA2-abs) and **(B)** using normalized sEMG-RMS amplitudes (CoA2-norm), presented as mean with 95% confidence interval. Calculated for three coactivation muscle pairs: tibialis anterior/gastrocnemius medialis, tibialis anterior/soleus, and rectus femoris/hamstring medialis, for each of the six gait phases (named in the bottom line). The agonist in the coactivation index muscle pair is indicated for each gait phase below each subplot. TA, tibialis anterior; GM, gastrocnemius medialis; SOL, soleus; RF, rectus femoris; HM, hamstring medialis. ^*^Significant group differences (*p* < 0.05), °*p* < 0.1.

Both absolute (column A, Figure 4) and normalized (column B, Figure 4) CoA1 values (CoA1-abs and CoA1-norm, respectively) were, in general, higher than 50% for both the TD and CP groups. The CoA1 values in RF/HM were relatively high and often ~100% (Figure 4), indicating equal activity of the agonist and antagonist muscles. Absolute CoA2-values (CoA2-abs, column A, Figure 5) were higher than 100% in three and six of 18 muscle pairs and gait phases in the TD and CP group, respectively, indicating higher activity of the antagonist than the agonist muscles. For normalized CoA2 values (CoA2-norm, column B, Figure 5), this was only seen once in TD and in five of 18 muscle pairs and gait phases in the CP group.

For CoA2, only TA/GM CoA2-abs during midswing/terminal swing and TA/GM CoA2-norm during weight acceptance phase were normally distributed. The rest of the variables were log transformed, resulting in normally distributed residuals. For 27 of the 34 variables, the results were similar for the log-transformed analysis as the original analysis. For the seven remaining, *p* values from the log-transformed analysis are presented.

For CoA1-abs (column A, Figure 4), TA/GM was increased in the CP group compared to the TD group during terminal stance (*p* < 0.01), had a trend to be reduced in the CP group during initial swing (*p* = 0.1), and was similar for both groups in the other four phases. Tibialis anterior/SOL was increased in the CP group during weight acceptance (*p* = 0.02) and terminal stance (*p* < 0.01) and was similar between the groups in the other four phases. Rectus femoris/HM showed no significant differences between the TD and CP groups (all *p* > 0.1).

For CoA1-norm (column B, Figure 4), TA/GM was increased in the CP group compared to TD during weight acceptance (*p* < 0.001), and midswing/terminal swing (*p* = 0.002), and there was a trend toward an increase in terminal stance (*p* = 0.06). In the other three phases, there were no differences between the groups. Tibialis anterior/SOL was increased in the CP group during weight acceptance (*p* < 0.001), there was a trend towards an increase in terminal stance (*p* = 0.08), and was similar for the groups in the other four phases. Rectus femoris/HM had a trend to be increased in the CP group during terminal stance (*p* = 0.05), and the increase was significant during preswing (*p* = 0.004). In the other four gait phases, there were no differences between the groups.

For CoA2-abs (column A, Figure 5), TA/GM was increased in the CP group compared to TD during terminal stance (*p* = 0.002), but was similar between the groups in the other five gait phases. Tibialis anterior/SOL was increased in the CP group during weight acceptance phase (*p* = 0.03) and during terminal stance (*p* = 0.01) but were similar between the groups in the other four phases. A borderline significant decrease was seen in the CP group for RF/HM during weight acceptance phase (*p* = 0.08), but no differences between the TD and CP groups were seen for the rest of the gait phases.

For CoA2-norm (column B, Figure 5), TA/GM was increased in the CP group compared to TD during weight acceptance phase (*p* < 0.001) and during midswing/terminal swing (*p* = 0.002). In the four gait phases in between, there were no differences between the groups. Tibialis anterior/SOL was increased in the CP group during weight acceptance phase (*p* < 0.001), and there was a trend toward an increase during terminal stance (*p* = 0.09). There were no differences between groups in the other four gait phases. Rectus femoris/HM had a borderline significant increase in the CP group during terminal stance (*p* = 0.05). During preswing, the increase in the CP group was significant (*p* = 0.004). In the remaining gait phases, there were no differences between the TD and CP groups.

## Discussion

The aim of this article was to investigate the effect of sEMG normalization on the interpretation of muscle activity and coactivation. Therefore, differences in muscle activity and coactivation indices between the healthy legs of TD children and the affected legs of ambulatory children with CP were examined using both absolute and normalized sEMG-RMS amplitudes (sEMG-RMS-abs and sEMG-RMS-norm, respectively). A secondary aim was to evaluate differences between two indices for calculating the coactivation index.

### Muscle Activity

Our results showed that normalization did not affect the average muscle activity pattern during gait within the TD or CP group but affected the between-subject variability within the groups and the difference in muscle activity between groups. Moreover, muscle activity deviations of the CP group from TD varied across the five investigated muscles.

Normalization of sEMG amplitudes is used to reduce between-subject variability caused by nonphysiological factors in order to compare physiological differences between muscles, individuals or sessions (16, 22, 31). However, it has been argued that as a consequence of normalizing the clinically relevant physiological variability could also be reduced (31). Our results show that the large between-subject variability seen in sEMG-RMS-abs during one to two specific gait phases is entirely equalized after normalization (Figure 3). This was seen in both groups. In the TD group, large between-subject variability in sEMG-RMS-abs was seen during weight acceptance phase for TA and SOL and during terminal stance for GM. In the CP group, TA, GM, SOL, and RF all showed large variability during weight acceptance phase only. The remainder of the gait cycle (~85%) showed low variability in sEMG-RMS-abs, suggesting low nonphysiological between-subject variability. This may indicate that the children have clinically relevant variation in muscle activity during some gait phases, which are diminished by normalization.

The overall muscle activity pattern in both groups was very similar across the two approaches of handling the sEMG data, because the gait phases with high and low amplitudes barely changed (Figure 3). This is in accordance with previous research evaluating the methods of sEMG normalization on healthy controls (18–20) and patients with stroke (19, 20). However, the deviations of the CP group from TD varied between the two approaches applied in this article. Increased sEMG-RMS-abs for CP in one gait phase, for instance, changed to reduced amplitude after normalization in another gait phase, or the other way around. Specifically, as seen in Figure 3 and quantified in Table 3, TA showed some reduced activity in the CP group compared to TD from initial swing to early weight acceptance phase for both sEMG-RMS-abs and sEMG-RMS-norm. However, these deviations reached statistical significance in different phases for the two approaches (both swing phases for sEMG-RMS-abs, whereas only the midswing/terminal swing for sEMG-RMS-norm, in addition to bordeline significance at weight acceptance phase; Table 3). Moreover, the TA amplitude during preswing seemed somewhat increased in the CP group but was only significantly increased after normalization. This could be a physiological compensation for the reduced activity during swing, or a result of normalization.

In the TD group, GM and SOL were especially active during three gait phases: the end of weight acceptance phase, terminal stance, and initial swing (Figure 3). During the end of weight acceptance, GM and SOL seemed somewhat increased in the CP group. However, they were significantly increased only after normalization. During terminal stance, GM and SOL activity in the CP group was decreased using sEMG-RMS-abs, but after normalization only GM was still significantly decreased. Similarly, during initial swing, GM and SOL activity in the CP group was decreased using sEMG-RMS-abs, but only SOL remained significantly decreased after normalization. In the CP group, RF activity was increased using sEMG-RMS-abs during weight acceptance and possible during midstance, but there were no differences between the groups for the last four gait phases. After normalization, the amplitudes were decreased for the CP group from terminal stance to midswing/terminal swing, but no differences were seen for the first two phases.

Despite these alterations in deviating amplitudes of the CP group from TD, in a clinical perspective, evaluating the overall muscle activity pattern may often be more essential in detecting phasic abnormalities rather than relative to TD amplitudes (22). Our results showed that the overall picture of the muscle activity pattern within each group did not seem to be so different between absolute and normalized sEMG amplitudes. However, details can be of clinical relevance. To only base interpretations on absolute or normalized sEMG could have consequences for treatment prescriptions. Should we, for instance, interpret the results as mainly overactivity of the calf muscles (GM and SOL) during weight acceptance and treat with botulinum toxin A, or as reduced activity during terminal stance and treat with strength training?

### Muscle Coactivation

There are several approaches for calculating the coactivation index, and in this article, we have looked closer at two indices commonly used. Our results showed statistically increased coactivation indices in the CP group compared to TD in three and four of 18 investigated muscle pairs and gait phases using absolute (CoA1-/CoA2-abs) and normalized (CoA1-/CoA2-norm) sEMG values, respectively. Although the two indices showed the same significant deviations in the CP group from TD, the values in CoA2 (Figure 5) were in general substantially higher than CoA1 values (Figure 4). Additionally, the between-subject variability was greater in CoA2 compared to CoA1, and as the variables of CoA2 had to be log transformed prior to analysis, CoA1 may therefore be more easily used when testing group differences. Hence, the following paragraphs will be based on CoA1.

In phases where there is low agonist activity and already very high coactivation indices in TD, even higher indices in CP cannot be expected. The TD group show clear agonist sEMG burst during weight acceptance, initial and midswing/terminal swing for TA, during terminal stance for SOL and GM and thus low coactivation indices (<75%) for both absolute and normalized values. Our findings indicate increased coactivation index in the CP group in 60% of these gait phases. These increased coactivation indices are in line with expectations and previous studies evaluating coactivation during walking in children with CP (9, 12).

However, there is no general agreement on the role of the coactivation in populations with neurological disorders, beyond abnormal levels that have been reported (15, 32). Potential explanations for our increased coactivation indices will be discussed below. During weight acceptance, the calf muscles–based coactivation indices (TA/GM and TA/SOL) were increased in the CP group for both CoA1-abs and CoA1-norm (although not statistically significant for TA/GM with CoA1-abs). For CoA1-abs, neither the agonist TA was decreased, nor the antagonist calf muscles (GM and SOL) increased, but for CoA1-norm, the antagonist calf muscles were increased in addition to somewhat decreased agonist TA. Using CoA1-norm, it seems that at least some of the children with CP showed increased index due to increased coactivation of the calf muscles. During terminal stance, however, the increased coactivation index accompanied by a largely reduced agonist calf muscle activity without increased antagonistic TA activity weakens the hypothesis of increased TA coactivation. Similarly, the increased coactivation index for the normalized TA/GM muscle pair during midswing/terminal swing was accompanied by reduced agonist TA activation and not increased antagonistic GM activity. Likewise, the increased coactivation index of the normalized RF/HM muscle pair during terminal stance and preswing was not accompanied by increased antagonistic HM, but by decreased agonistic RF activity. It is difficult to know if increased coactivation is due to excessive antagonist activity or due to muscle weakness in the agonist, without any knowledge of the underlying muscle activity. Moreover, a coactivation index of 100% represents equal activity of the agonist and antagonist muscle but does not say anything about the amount of activity. Both muscles could potentially be highly active or somewhat active, and the latter with a slight increase in antagonist muscle activity would lead to a highly increased coactivation index. To decide whether the increased coactivation index is of clinical relevance, it is crucial to consider the underlying muscle activity at the same time.

Additionally, the interpretation of the coactivation index is closely related to the handling of the sEMG data. Using absolute or normalized sEMG-RMS amplitudes in the calculations gives different pictures of which gait phases the CP group deviate from TD, which emphasizes the complexity of the coactivation index.

## Conclusion

This article showed that the interpretation of muscle activity and coactivation was affected by normalization approach when evaluating group differences. Although the overall muscle activity pattern did not differ between absolute and normalized sEMG-RMS amplitudes, normalization eliminated variability that could be interpreted as physiological variation within the children and deviating sEMG-RMS amplitudes were found in different phases after normalization. Taken together, these results emphasize the importance of being able to use absolute sEMG-RMS amplitudes in addition to the dynamic peak normalized values and to have knowledge about the underlying physiology in order to interpret sEMG data.

When interpreting the coactivation index, it is important to be aware of the methodological approach in order to understand the origin and function, before drawing conclusions on abnormal coactivation levels and making comparisons between different studies. Our findings suggest that increased coactivation index may be explained by other factors than excessive antagonist coactivation, such as the inability to sufficiently activate the agonist.

Because we do not know the truth, we cannot conclude whether to normalize the data and recommend considering both absolute and normalized data for a complete interpretation. However, future research should relate to functional outcomes, to better answer whether absolute or normalized sEMG-RMS amplitudes are favorable in the interpretations of altered muscle activity and coactivation index.

## Data Availability Statement

The datasets generated for this study will not be made publicly available due to Norwegian legislation. Questions regarding the datasets can be sent to the corresponding author.

## Ethics Statement

The studies involving human participants were reviewed and approved by Regional Committee for Medical and Health Research Ethics in Middle Norway (REK Central). Written informed consent to participate in this study was provided by the participants' legal guardian/next of kin.

## Author Contributions

YG, SB, and KR contributed conception and design of the study. YG and KR performed the statistical analysis. YG wrote the first draft of the manuscript. All authors contributed to manuscript revision, read and approved the submitted version.

## Conflict of Interest

The authors declare that the research was conducted in the absence of any commercial or financial relationships that could be construed as a potential conflict of interest.
